# Fracture of Neck of Femur, and Autopsy

**Published:** 1849-10

**Authors:** Darwin Colvin


ART. III.—*Fracture of Neck of Femur, and Autopsy.* Reported to Dr. Hamilton, by Darwin Colvin, M. D.

T. B. S., aged 38 years, of intemperate habits, much emaciated, and having for the last year or two suffered from hepatic disturbance to such a degree as that his skin has been constantly of a deep yellow color, received an injury, Aug. 21, 1849, while in a state of intoxication. No one was present at the time of the accident, and it is not ascertained how it occurred. I saw him several hours afterward. The toes were turned out, and the leg shortened one inch and a half. The thigh was enormously swollen, and the whole pelvic region ecchymosed, with evidences of contusion, especially over the great trocanter. The trocanter could not be felt. Having made extension and rotation, with the assistance of my father, and crepitus being manifest, we determined that it was a fracture of the neck of the femur.

The limb was placed in a comfortable situation, with a view to the reduction of the inflammation.

On the 23d delirium tremens supervened, and on the 2d inst. he died, twelve days after the occurrence of the fracture.

*Autopsy,* eight hours after death:—

The neck of the femur was broken half an inch from the root of the trocanter major: the upper end of the lower fragment was comminuted, and the trocanter itself was completely separated and drawn up under the gluteus maximus. The head of the femur was partially removed from the acetabulum. The spongy structure of the bones was yellow, and nearly of the same color as the skin.

